# Construction of miRNA-mRNA regulatory network indicates potential biomarkers for primary open-angle glaucoma

**DOI:** 10.1186/s12920-023-01698-2

**Published:** 2023-11-08

**Authors:** Xiaoyu Zhou, Feng Zhang, Xinyue Zhang, Dengming Zhou, Yang Zhao, Baihua Chen, Xuanchu Duan

**Affiliations:** 1 Aier Glaucoma Institute, Changsha Aier Eye Hospital, Changsha, Hunan Province China; 2 The Second Xiangya Hospital, Central South University, Changsha, Hunan Province China; 3 The Third Xiangya Hospital, Central South University, Changsha, Hunan Province China; 4 Aier School of Ophthalmology, Central South University, Changsha, Hunan Province China

**Keywords:** Primary open‑angle glaucoma, miRNA-mRNA regulatory network, Biomarker

## Abstract

**Background:**

Trabecular meshwork (TM) dysfunction-induced elevation of intraocular pressure has been identified as the main risk factor of irreversible optic nerve injury in Primary open‑angle glaucoma (POAG). Increasing evidences suggest that microRNA (miRNA) plays a vital role in the pathogenesis of POAG. This study aims to construct a miRNA-mRNA regulatory network and identify biomarkers for POAG.

**Methods:**

miRNAs and mRNAs expression profiling of TM samples from controls and POAG patients were assessed through microarray analysis. Target genes of differentially expressed miRNAs (DEmiRNAs) were predicted by miEAA and miRNet. Then GO and KEGG pathway analysis of differentially expressed mRNAs (DEmRNAs) were performed. PPI of top 30 hub genes was identified and miRNA-mRNA network was established by STRING database and Cytoscape software. GSE27276 and GSE105269 datasets were used to verify the expression of hub genes and to predict potential biomarkers in TM and aqueous humor (AH) for POAG, respectively. Finally, GSEA analysis was conducted to estimate the main signaling pathway of POAG pathogenesis.

**Results:**

A total of 29 up-regulated and 7 down-regulated miRNAs, 923 up-regulated and 887 down-regulated mRNAs were identified in TM of POAG compared with controls. Target genes and DEmRNAs were mainly enriched in nitric oxide biosynthetic process, vasopressin-regulated water reabsorption, and so on. Through miRNA-mRNA network construction, top 30 hub genes were regulated by 24 DEmiRNAs. 8 genes were aberrantly expressed in dataset GSE27276. 3 genes (*CREB1*, *CAPZA2*, *SLC2A3*) and 2 miRNAs (*miR-106b-5p*, *miR-15a-5p*) were identified as potential biomarkers for POAG in TM and AH, respectively. GSEA analysis revealed that these 3 genes modulated POAG through different pathways.

**Conclusion:**

In this study, construction of miRNA-mRNA network and identification of biomarkers provide a novel insight into the pathogenesis, early diagnosis and treatment for POAG.

**Supplementary Information:**

The online version contains supplementary material available at 10.1186/s12920-023-01698-2.

## Background

With the population aging process, the prevalence of glaucoma has yearly increased, making glaucoma become the second leading cause of blindness following cataract. It is estimated that by 2040, the number of glaucoma patients will increase to 112 million [1]. Glaucoma, characterized by optic atrophy and visual field defect, is a multifactorial disease. Elevated intraocular pressure (IOP) caused by increased aqueous humor (AH) outflow resistance has been identified as the main risk factor for glaucoma onset and progression to blindness [2]. Primary open‑angle glaucoma (POAG) is the most common subtype of glaucoma, and accounts for approximately 74% of all populations with glaucoma [3]. The progressive dysfunction of trabecular meshwork (TM) cells and the imbalance between the synthesis and decomposition rate of extracellular matrix (ECM) lead to the increase of ECM deposition in aqueous humor outflow channel, which is the main cause for the increase of aqueous outflow resistance [2]. The exact pathophysiological mechanism of outflow resistance warrants further investigation.

MicroRNA (miRNA) is a non-coding single stranded small RNA that regulates complementary mRNA at the post transcriptional level in eukaryotes. As an important regulatory factor, miRNAs play an important role in the occurrence and development of POAG, and are widely involved in the biological process of regulating POAG related genes. MiRNA is associated with maintaining AH homeostasis, changing the structure of TM and ECM, and retinal ganglion cells (RGC) apoptosis [4]. MiRNA can affect the structure of TM by regulating non-housekeeping genes expression [5]. Previous study has transfected trabecular meshwork cells with *miR-29b* mimic and found that it is associated with the deposition and recombination of ECM [6]. *MiR-24* can negatively regulate the protease *FURIN*, the activator of *TGF-β*, thus affecting the ECM metabolism of TM [7]. In addition, miRNA exist as exosomes, apoptotic bodies, protein/miRNA complexes and other forms in AH, which may maintain the shape of the anterior chamber and the pressure of AH by regulating the target genes of tissues related to the anterior chamber (such as TM). For example, *miR-184* in AH has been shown to regulate the phagocytosis of TM [8]. At present, many studies have explored the potential of miRNA as a possible biomarker in ophthalmic diseases. A bioinformatic analysis study has constructed a competing endogenous RNA (ceRNA) transcriptional regulatory network and identified biomarkers in AH of POAG [9]. And our previous study has revealed the important role of long non-coding RNAs and mRNAs interactions in POAG [10]. Study on the role of miRNA in POAG is of great significance for the early diagnosis and treatment of glaucoma.

In this study, we performed a combined multiomics analysis of miRNA and mRNA expression in TM and AH tissues of POAG. We aimed to construct a miRNA-mRNA regulatory network, reveal potential signaling pathway regulatory mechanisms and identify biomarkers for POAG. This may contribute to clinical prevention, early diagnosis and individualized treatment of POAG.

## Methods

### Ethical statement

All experiments associated with human participants in this study were approved by the Ethics Committee of the Second Xiangya Hospital of Central South University and the 1964 Helsinki declaration and its later amendments or comparable ethical standards. Informed consent was obtained from all individual participants enrolled in the study, in Ethics approval and consent to participate section.

### Microarray

This study enrolled 23 TM tissues from patients with POAG and 12 TM tissues from healthy controls who donated corneas in the Second Xiangya Hospital, Central South University. The lncRNA, miRNA and mRNA expression profiles in TM of POAG were determined through microarray analysis. The specific protocol has been described in a previously published article [10]. The tiny TM tissue from one glaucoma patient undergoing trabeculectomy was not enough for one RNAseq test. So we mixed 5–6 single TM tissue for one test. TM tissue from volunteer who donated their cornea was more complete, but still not enough for one test. So we mixed 3 single TM tissue for one test. Thus, we got 4 tests for each group.

### Availability of data and materials statement

The datasets generated and/or analysed during the current study are available in the Gene Expression Omnibus public database GSE138125 (mRNA-seq), GSE231760 (miRNA-seq), GSE27276 (genome-wide expression in TM) and GSE105269 (miRNA-seq in AH) (https://www.ncbi.nlm.nih.gov/).

### Analysis of differentially expressed miRNAs and mRNAs

Volcano maps and heat maps showing analysis of differentially expressed miRNAs (DEmiRNAs) and mRNAs (DEmRNAs) and principal component analysis (PCA) were generated using ggplot2 package (3.3.3) in R (3.6.3). The analysis threshold was used: |logFC|> 2 for miRNAs, |logFC|> 1 for mRNAs and adjusted *P* < 0.05. LogFC > 0 represents up-regulated genes; logFC < 0 represents down-regulated genes. |logFC| represents the fold change of mRNA or miRNA expression levels. So, the threshold of |logFC| varies from different studies objectives or data types. We got a huge number of differentially expressed miRNAs and their target mRNAs when we used |logFC|> 1 for both miRNAs and mRNAs. This makes it difficult to screen hub genes. Meanwhile, when we used |logFC|> 2, we got a small number of differentially expressed mRNAs, which makes it difficult to perform GO, KEGG or GSEA analysis. Therefore, we had to use different thresholds of |logFC|.

### GO and KEGG functional enrichment analysis

Gene Ontology (GO) annotation and Kyoto Encyclopedia of Genes and Genomes (KEGG) pathway functional enrichment analyses were performed to annotate the biological function of DEmRNAs and target genes of DEmiRNAs [11–13]. The clusterProfiler package (3.14.3) and ggplot2 package (3.3.3) in R (3.6.3) were used for enrichment analysis and visualization; org.Hs.eg.db (3.10.0) package was used for ID conversion; GOplot package (1.0.2) was used to calculate zscore [14]. The analysis threshold was used: adjusted *P* < 0.05.

### Prediction of downstream target genes of DEmiRNAs

miEAA database (https://ccb-compute2.cs.uni-saarland.de/mieaa2/) and miRNet database (https://www.mirnet.ca/), providing information of miRNAs and its target genes interactions, were used to predict the downstream target genes of DEmiRNAs. Then we use the Venn diagrams to analyze the overlap between these two databases. The miRNA-target gene network was generated by the website of miRNet. We further analyze the overlap of target genes of DEmiRNAs and DEmRNAs by Venn diagrams.

### Construction of miRNAs-mRNAs regulatory network

Search Tool for the Retrieval of Interacting Genes/Proteins (STRING) online database (http://stringdb.org/) was used to construct the protein–protein interaction (PPI) network of DEmiRNAs target genes. The visualization was performed by CytoHubba plugin of Cytoscape (3.9.0). Top 30 hub nodes of genes were selected by topological algorithm ranking degree method. The miRNAs-mRNA network was also established by Cytoscape according information of miRNAs and its target genes interactions in miRNet database. Sankey diagram was generated by ggalluvial package in R (3.6.3).

### Verification of key genes expression by GSE27276 dataset

We downloaded the GSE27276 dataset from the GEO database to verify the expression of top 30 hub genes in TM identified by PPI network. Student’s t-test was used to test for the significance between POAG and controls. *P* < 0.05 was considered statistically significant.

### Prediction of biomarkers in TM and AH

The ROC curves were used to predict the potential of key genes identified above as biomarkers for POAG through pROC (for analysis) and ggplot2 (for visualization) package in R (3.6.3). We also downloaded the GSE105269 dataset from the GEO database to construct diagnostic ROC curves of miRNAs related to screened hub genes in AH of POAG.

### GSEA enrichment analysis

We divided samples of subjects enrolled in GSE27276 dataset into high expression group and low expression group according to the absolute expression levels of *CREB1*, *CAPZA2* and *SLC2A3* in these samples. Then clusterProfiler and ggplot2 package in R (3.6.3) were used for Gene Set Enrichment Analysis (GSEA). Reference gene set: c2.cp.v7.2.symbols.gmt [Curated]. Gene set database: MSigDB Collections (database hyperlinks).

## Results

### Differential expression of miRNAs and mRNAs in TM depicts the transcriptional characteristics of POAG

**Fig. 1:**
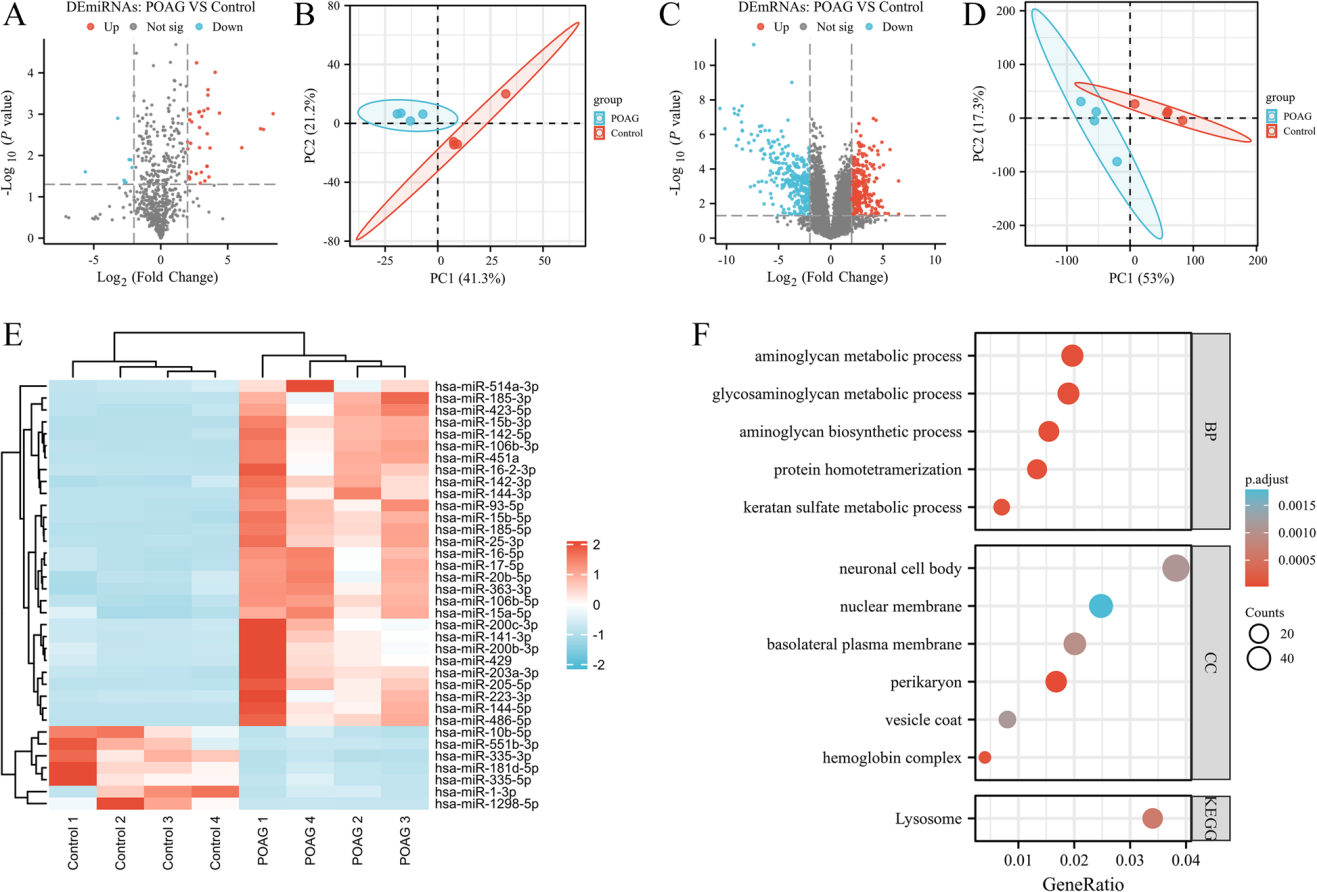
The differentially expressed miRNAs and mRNAs in TM of POAG. **A**, **C** The miRNAs and mRNAs microarray expression profiling of controls and POAG patients in TM tissue is displayed by volcano map. The horizontal dotted lines and vertical dotted lines indicate limits for *P* values and fold change, respectively (|logFC|> 2 for miRNAs, or > 1 for mRNAs, and adjusted *P* < 0.05). **B**, **D** Data variability between control and POAG samples is displayed by PCA. **E** Heat map of the DEmiRNAs between controls and POAG patients in TM. Each small square represents the color depth corresponding to the expression value of the i-th row gene corresponding to the j-th column sample, which is converted by zscore. Red bars represent up-regulated DEmiRNAs, blue bars represent down-regulated DEmiRNAs. **F** GO annotation and KEGG pathway functional enrichment analyses of DEmRNAs by dotplot in TM for POAG

The clinical characteristics of patients included were displayed in Table 1. Table 2 showed the datasets from GEO database we used in this study. 29 up-regulated and 7 down-regulated miRNAs (|logFC|> 2, adjusted *P* < 0.05), 923 up-regulated and 887 down-regulated mRNAs (|logFC|> 1, adjusted *P* < 0.05) were identified in TM of POAG compared with controls (Fig. 1A, C). PCA of the data cleanly distinguished POAG from controls (Fig. 1B, D). Heatmap displayed expression levels of miRNAs in various samples (Fig. 1E, Table 3). We performed GO annotation and KEGG pathway functional enrichment analyses to explore the potential role of DEmRNAs and related signaling pathways in POAG (Fig. 1F). 5 GO terms from the categories of biological process (BP) were enriched, including aminoglycan biosynthetic process, protein homotetramerization, glycosaminoglycan metabolic process, aminoglycan metabolic process and keratan sulfate metabolic process. 6 GO terms of cellular component (CC) were enriched, including perikaryon, hemoglobin complex, basolateral plasma membrane, neuronal cell body, vesicle coat and nuclear membrane. Only one KEGG pathway, lysosome, was enriched in DEmRNAs of TM from POAG.

**Table 1:** Clinical characteristics of participants enrolled in this study

Characteristics	Control (*n* = 12)	POAG (*n* = 23)
Age, years, mean ± SD	49.83 ± 10.16	50.43 ± 9.72
Sex		
Male	7	10
Female	5	13

**Table 2:** Datasets enrolled in this study

GEO datasets	Platform	Tissue	Control (n)	POAG (n)
GSE138125	GPL18573	TM	4	4
GSE231760	GPL21827	TM	4	4
GSE27276	GPL2507	TM	19	17
GSE105269	GPL24158	AH	11	12

**Table 3:** Differentially expressed miRNAs

Up-regulated				Down-regulated
miR-106b-3p	miR-15a-5p	miR-185-5p	miR-25-3p	miR-1-3p
miR-106b-5p	miR-15b-3p	miR-200b-3p	miR-363-3p	miR-10b-5p
miR-141-3p	miR-15b-5p	miR-200c-3p	miR-423-5p	miR-1298-5p
miR-142-3p	miR-16–2-3p	miR-203a-3p	miR-429	miR-181d-5p
miR-142-5p	miR-16-5p	miR-205-5p	miR-451a	miR-335-3p
miR-144-3p	miR-17-5p	miR-20b-5p	miR-486-5p	miR-335-5p
miR-144-5p	miR-185-3p	miR-223-3p	miR-514a-3p	miR-551b-3p
miR-93-5p				

### Identification of the key target mRNAs in TM indicates the signaling pathways of POAG

**Fig. 2:**
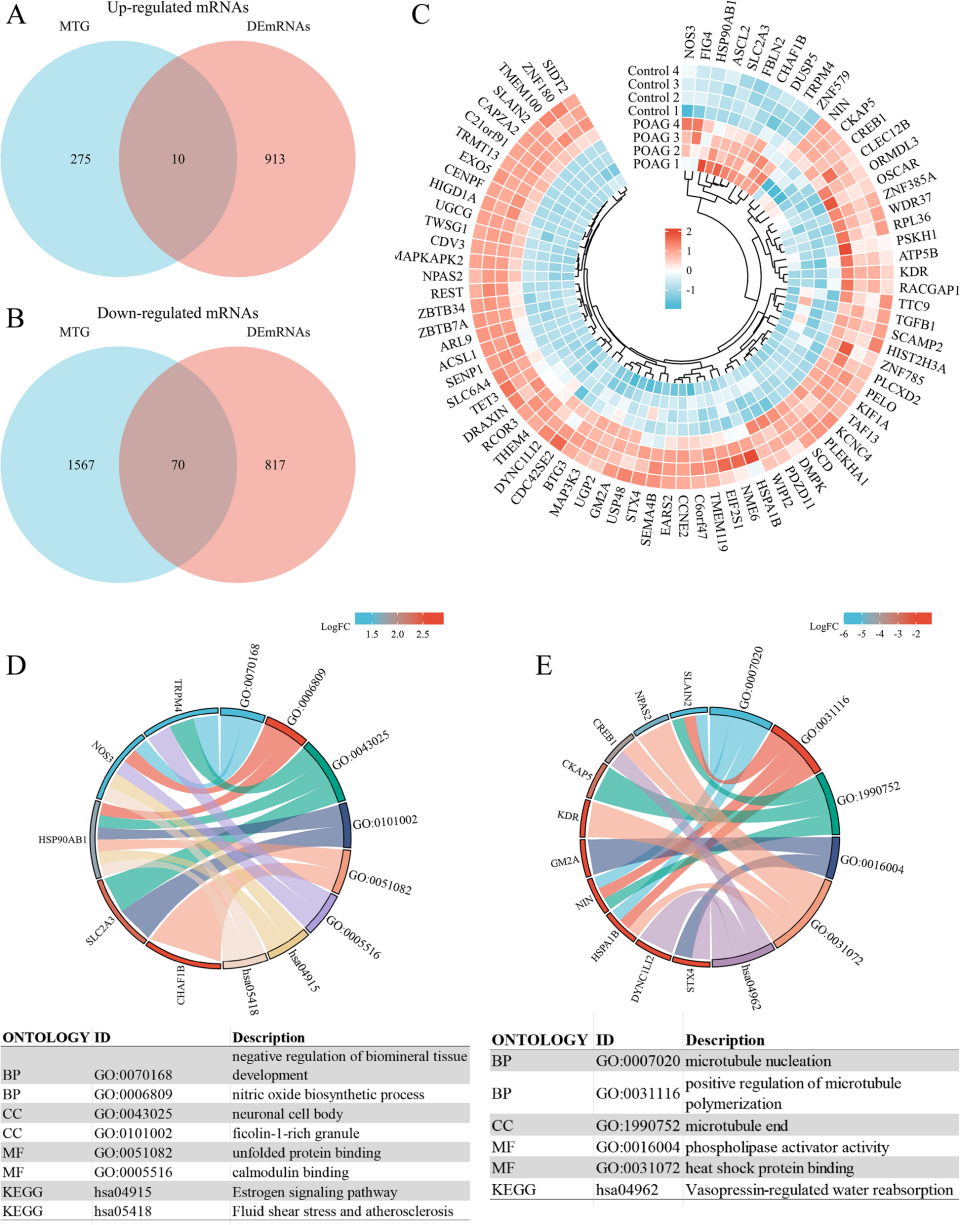
Identification of the key target mRNAs in TM of POAG. **A**, **B** Venn diagrams indicate overlaps of DEmiRNAs target genes (MTG) and DEmRNAs in TM for POAG. **C** Circos heat map of potential DEmiRNAs target DEmRNAs between controls and POAG patients in TM. Red bars represents up-regulated DEmRNAs, blue bars represents down-regulated DEmRNAs. **D**, **E** GO annotation and KEGG pathway functional enrichment analyses (combined with LogFC) of DEmiRNAs target DEmRNAs

Firstly, we predicted the downstream target genes of DEmiRNAs by the overlap of miEAA and miRNet databases. 1637 target genes of up-regulated miRNAs and 285 target genes of down-regulated miRNAs were identified (Fig. S1). Then we took the intersection of miRNAs target genes (MTG) and DEmRNAs in TM of POAG, and obtained 10 up-regulated target mRNAs and 70 down-regulated target mRNAs (Fig. 2A, B). The circos heat map displayed the expression variations of DEmiRNAs-targeted DEmRNAs in TM between controls and POAG patients (Fig. 2C). GO annotation and KEGG pathway functional enrichment analyses were performed in combination with logFC of target mRNAs (Fig. 2D, E). BP analysis showed that up-regulated target mRNAs and down-regulated mRNAs were significantly enriched in negative regulation of biomineral tissue development, nitric oxide biosynthetic process and microtubule nucleation, positive regulation of microtubule polymerization, respectively. CC analysis revealed that up-regulated target mRNAs and down-regulated mRNAs were mainly enriched in neuronal cell body, ficolin-1-rich granule and microtubule end, respectively. Molecular function (MF) analysis displayed that up-regulated target mRNAs and down-regulated mRNAs were particularly enriched in unfolded protein binding, calmodulin binding and phospholipase activator activity, heat shock protein binding, respectively. KEGG pathway proved that up-regulated target mRNAs and down-regulated mRNAs were enriched in the estrogen signaling pathway, fluid shear stress and atherosclerosis and vasopressin-regulated water reabsorption, respectively.

### Construction of miRNA–mRNA network reveals molecular regulatory mechanism for POAG

**Fig. 3:**
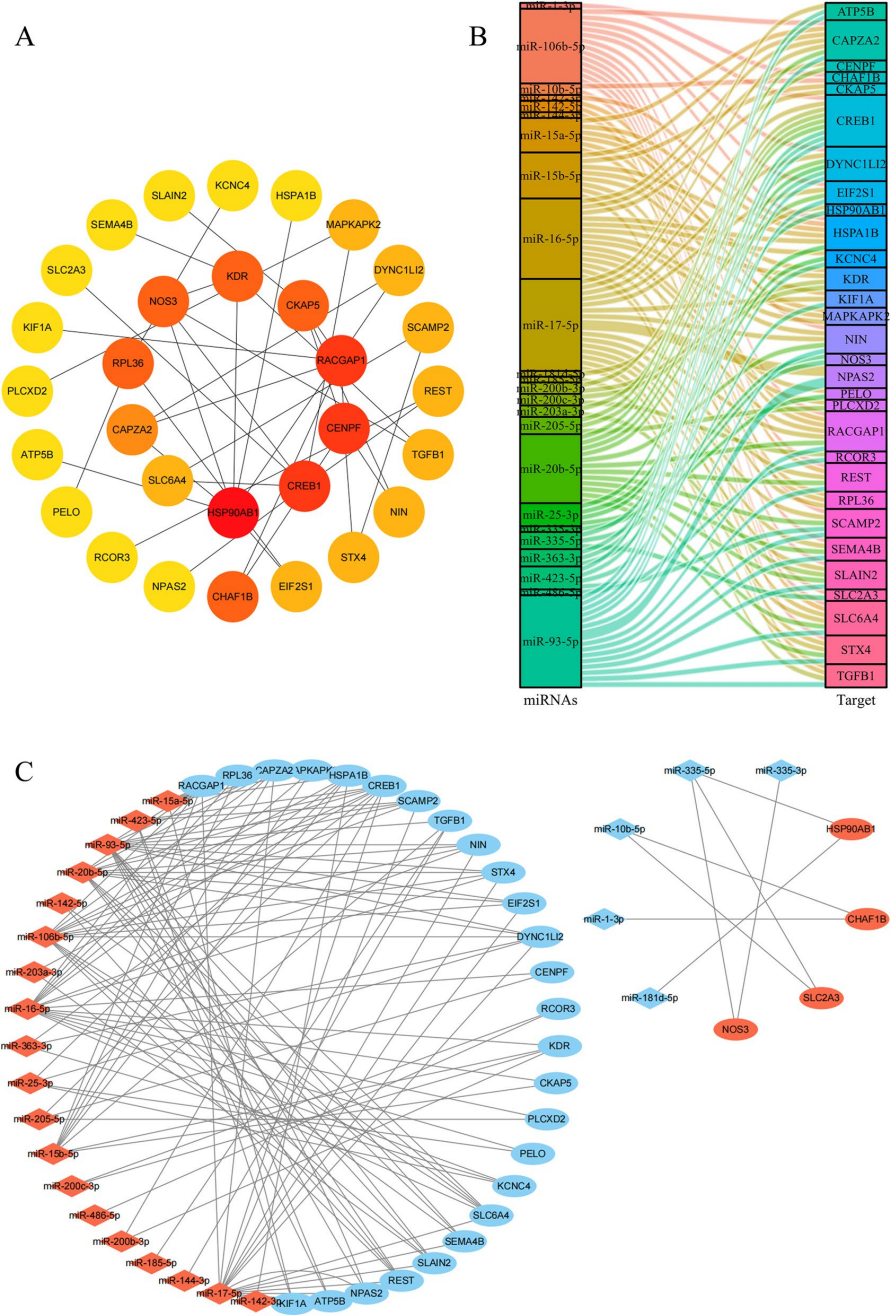
Construction of miRNA-mRNA regulatory network. **A** PPI network of the top 30 hub DEmiRNAs-targeted DEmRNAs. **B** Sankey diagram of the interactions among miRNAs regulating mRNAs. **C** The hub miRNA-mRNA regulatory network in TM of POAG. Red represents up-regulated mRNAs or miRNAs, blue represents down-regulated mRNAs or miRNAs. Ellipse represents mRNAs, diamond represents miRNAs

We established the PPI network for both up-regulated and down-regulated mRNAs targeted by DEmiRNAs using the STRING database. Then top 30 hub target mRNAs were analyzed by Cytoscape and cytoHubba plugin and were arrayed by degree (Fig. 3A and Table 4). Sankey diagram and miRNAs-mRNA network exhibited the interactions between DEmiRNAs and hub DEmRNAs (Fig. 3B, C and Table 5). 19 up-regulated miRNAs might restrain transcription of 26 downstream mRNAs. 5 down-regulated miRNAs might promote transcription of 4 downstream mRNAs.

**Table 4:** Top 30 hub genes

Up-regulated	Down-regulated				
HSP90AB1	CENPF	CAPZA2	DYNC1LI2	NPAS2	PELO
NOS3	CREB1	TGFB1	SCAMP2	REST	RCOR3
CHAF1B	RACGAP1	NIN	SLC6A4	ATP5B	
SLC2A3	KDR	STX4	SEMA4B	PLCXD2	
	CKAP5	EIF2S1	SLAIN2	KIF1A	
	RPL36	MAPKAPK2	HSPA1B	NPAS2

**Table 5:** miRNAs-mRNA interactions

miRNA	mRNA	miRNA	mRNA
miR-16-5p	CENPF	miR-15b-5p	KDR
miR-205-5p	CENPF	miR-16-5p	KDR
miR-106b-5p	CREB1	miR-200b-3p	KDR
miR-17-5p	CREB1	miR-200c-3p	KDR
miR-200b-3p	CREB1	miR-15b-5p	CKAP5
miR-203a-3p	CREB1	miR-16-5p	CKAP5
miR-205-5p	CREB1	miR-15a-5p	RPL36
miR-20b-5p	CREB1	miR-15b-5p	RPL36
miR-363-3p	CREB1	miR-16-5p	RPL36
miR-423-5p	CREB1	miR-106b-5p	CAPZA2
miR-93-5p	CREB1	miR-15a-5p	CAPZA2
miR-106b-5p	RACGAP1	miR-15b-5p	CAPZA2
miR-15a-5p	RACGAP1	miR-16-5p	CAPZA2
miR-15b-5p	RACGAP1	miR-17-5p	CAPZA2
miR-16-5p	RACGAP1	miR-20b-5p	CAPZA2
miR-17-5p	RACGAP1	miR-93-5p	CAPZA2
miR-20b-5p	RACGAP1	miR-144-3p	TGFB1
miR-93-5p	RACGAP1	miR-15b-5p	TGFB1
miR-106b-5p	STX4	miR-185-5p	TGFB1
miR-16-5p	STX4	miR-93-5p	TGFB1
miR-17-5p	STX4	miR-106b-5p	NIN
miR-20b-5p	STX4	miR-16-5p	NIN
miR-93-5p	STX4	miR-17-5p	NIN
miR-106b-5p	EIF2S1	miR-20b-5p	NIN
miR-17-5p	EIF2S1	miR-93-5p	NIN
miR-20b-5p	EIF2S1	miR-15a-5p	MAPKAPK2
miR-93-5p	EIF2S1	miR-15b-5p	MAPKAPK2
miR-106b-5p	SCAMP2	miR-16-5p	MAPKAPK2
miR-17-5p	SCAMP2	miR-106b-5p	DYNC1LI2
miR-20b-5p	SCAMP2	miR-17-5p	DYNC1LI2
miR-423-5p	SCAMP2	miR-20b-5p	DYNC1LI2
miR-93-5p	SCAMP2	miR-25-3p	DYNC1LI2
miR-106b-5p	SLC6A4	miR-363-3p	DYNC1LI2
miR-142-5p	SLC6A4	miR-93-5p	DYNC1LI2
miR-16-5p	SLC6A4	miR-106b-5p	SLAIN2
miR-17-5p	SLC6A4	miR-17-5p	SLAIN2
miR-20b-5p	SLC6A4	miR-203a-3p	SLAIN2
miR-93-5p	SLC6A4	miR-20b-5p	SLAIN2
miR-106b-5p	SEMA4B	miR-93-5p	SLAIN2
miR-17-5p	SEMA4B	miR-106b-5p	HSPA1B
miR-20b-5p	SEMA4B	miR-142-3p	HSPA1B
miR-93-5p	SEMA4B	miR-15a-5p	HSPA1B
miR-16-5p	PELO	miR-15b-5p	HSPA1B
miR-25-3p	PELO	miR-16-5p	HSPA1B
miR-200c-3p	RCOR3	miR-25-3p	HSPA1B
miR-486-5p	RCOR3	miR-17-5p	NPAS2
miR-17-5p	NPAS2	miR-93-5p	NPAS2
miR-93-5p	NPAS2	miR-106b-5p	REST
miR-16-5p	KCNC4	miR-142-5p	REST
miR-25-3p	KCNC4	miR-17-5p	REST
miR-363-3p	KCNC4	miR-20b-5p	REST
miR-15a-5p	KIF1A	miR-93-5p	REST
miR-17-5p	KIF1A	miR-181d-5p	HSP90AB1
miR-423-5p	KIF1A	miR-335-5p	HSP90AB1
miR-17-5p	ATP5B	miR-335-3p	NOS3
miR-423-5p	ATP5B	miR-335-5p	NOS3
miR-93-5p	ATP5B	miR-1-3p	CHAF1B
miR-16-5p	PLCXD2	miR-10b-5p	CHAF1B
miR-205-5p	PLCXD2	miR-10b-5p	SLC2A3
		miR-335-5p	SLC2A3

### Verification of key genes expression facilitating POAG

**Fig. 4:**
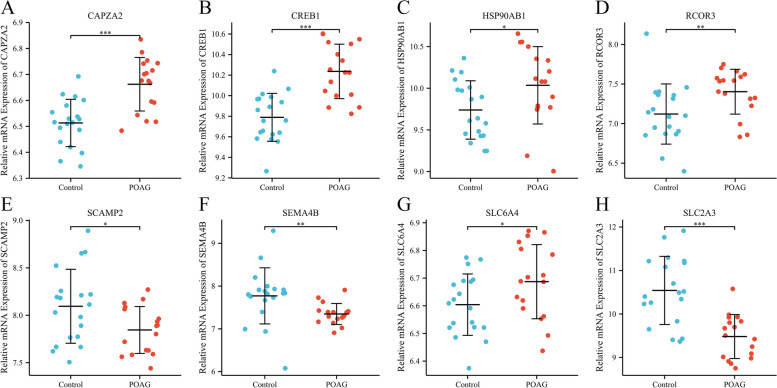
Identification of 8 hub genes displayed by expression levels from the GSE27276 dataset. *P < 0.05, **P < 0.01, ***P < 0.001

Based on the miRNA–mRNA network, expression levels of the top 30 hub genes in TM were validated in the GSE27276 dataset. 5 hub genes (*CAPZA2*, *CREB1*, *HSP90AB1*, *RCOR3*, *SLC6A4*) were up-regulated. 3 hub genes (*SCAMP2*, *SEMA4B*, *SLC2A3*) were down-regulated in TM of patients with POAG compared with that of controls (Fig. 4). Other genes showing no significant difference between controls and POAG patients were displayed in Fig. S2.

### Biomarkers of POAG

**Fig. 5:**
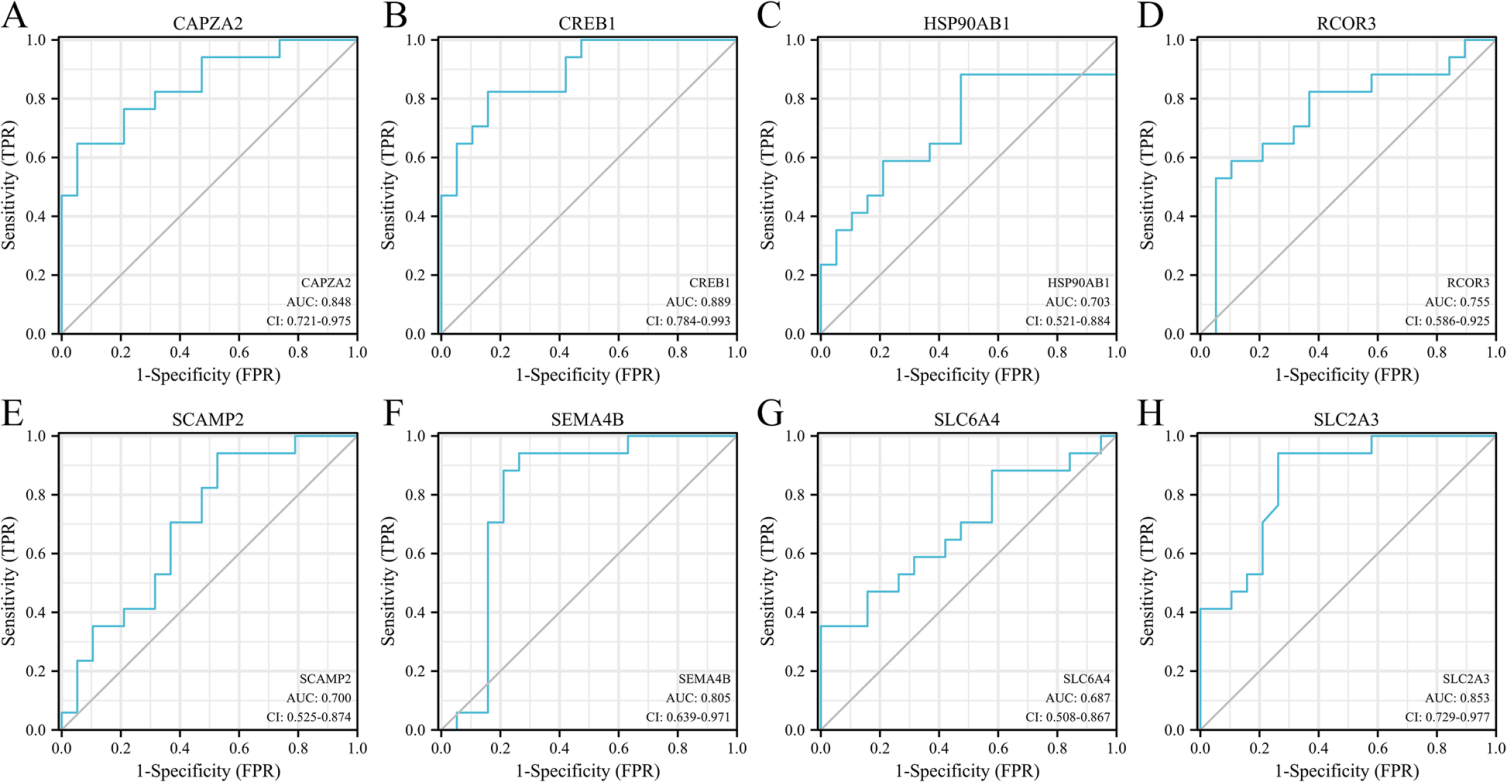
Identification of biomarkers for POAG in TM by ROC curves. AUC at 0.5 ~ 0.7 represents low accuracy, AUC at 0.7 ~ 0.9 represents moderate accuracy, AUC above 0.9 represents high accuracy

**Fig. 6:**
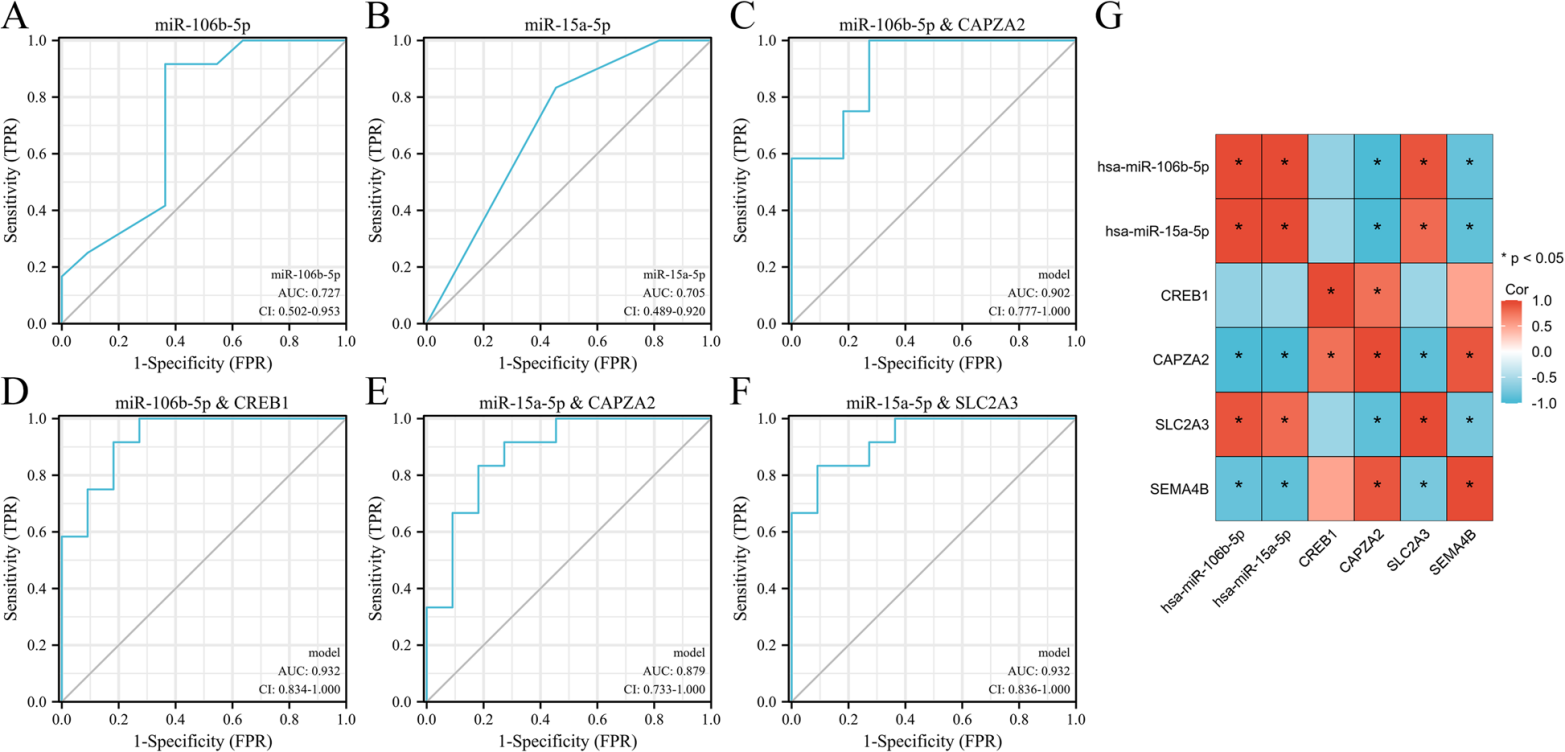
Identification of biomarkers for POAG in AH by ROC curves. **A**, **B** The potential of *miR-106b-5p* and *miR-15a-5p* in AH for identification of POAG from GSE105269 dataset. **C**-**F** The potential of *miR-106b-5p* and *miR-15a-5p* combined with their target gene for identification of POAG. AUC at 0.5 ~ 0.7 represents low accuracy, AUC at 0.7 ~ 0.9 represents moderate accuracy, AUC above 0.9 represents high accuracy

We established ROC curves to screen the biomarkers among key genes identified above as characteristics to distinguish POAG from non-glaucoma individuals (Fig. 5). *HSP90AB1*, *RCOR3*, *SCAMP2* and *SLC64A* (AUC < 0.8) showed poor ability to distinguish POAG from non-glaucoma individuals. While *CAPZA2*, *CREB1*, *SEMA4B* and *SLC2A3* (AUC > 0.8) exhibited fairly good accuracy as biomarkers. Given that biomarkers in TM are difficult to be used in clinical practices, we assessed the potential of miRNAs in AH as biomarkers for POAG from GSE105269 dataset. ROC curves showed that *miR-106-5p* and *miR-15a-5p* exhibited the highest accuracy (AUC = 0.727 and 0.705, respectively) as biomarkers among 12 DEmiRNAs corresponding to *CAPZA2*, *CREB1*, *SEMA4B* and *SLC2A3* (Fig. 6A, B and Fig. S3). Furthermore, we used miRNA-mRNA axis to establish a more stable biomarker system for POAG. ROC curves suggested that *miR-106-5p-CAPZA2*/*CREB1* axis and *miR-15a-5p-SLC2A3* axis (AUC > 0.9) exhibited excellent ability to distinguish POAG from non-glaucoma individuals (Fig. 6C-F). We performed correlation analysis of these biomarkers (Fig. 6G). *CREB1* is not correlated with *miR-106-5p* or *miR-15a-5p*. *CAPZA2* and *SEMA4B* are both negatively correlated with *miR-106-5p* and *miR-15a-5p*. *SLC2A3* is positively correlated with *miR-106-5p* and *miR-15a-5p*. It is worth noting that *SLC2A3* is not target gene of *miR-106-5p* or *miR-15a-5p*. Here, we combined them to predict POAG just because of their high AUC.

### Critical signaling pathways mediated by key genes in POAG

**Fig. 7:**
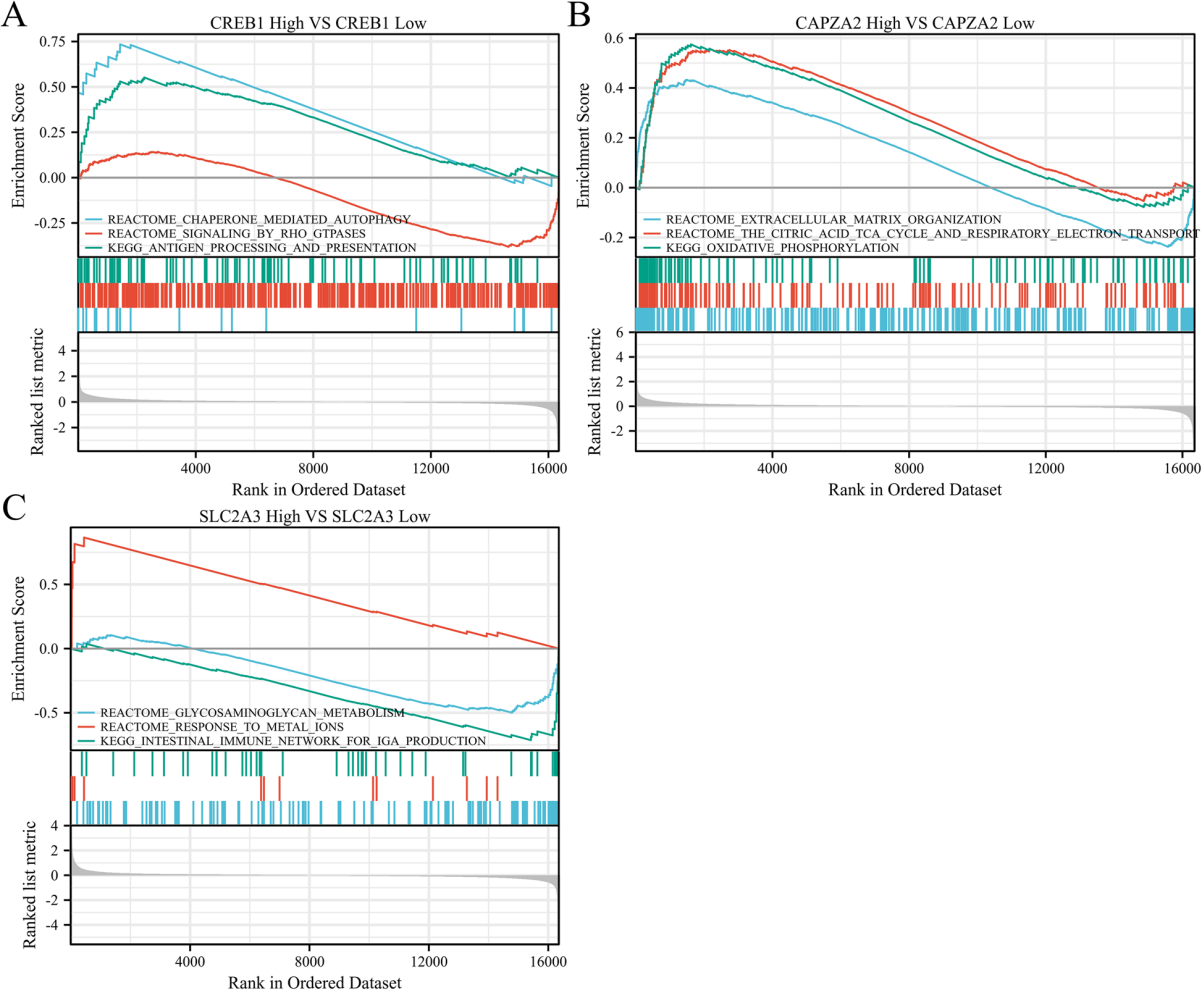
GSEA enrichment plots indicate signaling pathways mediated by up-regulated *CREB1*, *CAPZA2* and down-regulated *SLC2A3*

To investigate the signaling pathways mediated by key genes identified above in POAG, we performed GSEA enrichment analysis using data from GSE27276 dataset, as shown in Fig. 7. The results showed that chaperone mediated autophagy and signaling by Rho GTPases were enriched in the group of patients with up-regulated *CREB1* (Fig. 7A). Extracellular matrix organization, the citric acid TCA cycle and respiratory electron transport, oxidative phosphorylation were enriched in the group of patients with up-regulated *CAPZA2* (Fig. 7B). Response to metal ions were enriched in the group of patients with up-regulated *SLC2A3*. Glycosaminoglycan metabolism and intestinal immune network for IGA production were enriched in the group of patients with down-regulated *SLC2A3* (Fig. 7C). These results indicated a novel insight into pathogenesis and potential therapeutic target for POAG.

## Discussion

In the present study, we performed a combined multiomics analysis of miRNA and mRNA expression in TM and AH tissues of POAG. A total of 29 up-regulated and 7 down-regulated miRNAs were identified in TM of POAG compared with controls. Among up-regulated DEmiRNAs, *miR-141* may regulate TM cells and RGC apoptosis via *PI3K*/*Akt*/*mTOR* pathway [15]. *miR-200c* can modulate TM cell contraction and IOP [16]. And *miR-93* promotes oxidative stress and inhibits cytoactive of TM in POAG through suppressing *Nrf2* [17]. For down-regulated DEmiRNAs, *miR-1* and its target gene *MALAT1* are associated with lower risk and severity of normal-tension glaucoma (NTG) [18]. The down-regulation of *miR-1298* in glaucoma pathology contributes to *TGF-β2/Smad4* pathway and TM injury [19]. The reduced expression of *miR-335-3p* also promotes RGC apoptosis via regulating autophagy in glaucoma [20]. The *miR-486-5p* and *miR-143-3p* are the two most frequently dysregulated microRNAs. In our study, there was no significant statistical difference of *miR-143-3p* between the POAG and control groups (*P* = 0.079) in our dataset. The *miR-486-5p* was up-regulated in POAG compared with control (*P* < 0.05, logFC = 7.45). However, its target gene RCOR3 showed poor ability to distinguish POAG from non-glaucoma individuals (AUC = 0.755). It is worth noting that most of these DEmiRNAs were studied more in cancer biology. Further studies are required to explore the role of miRNAs in POAG.

The complicated pathogenesis limited the development of glaucoma diagnosis and treatment, leading to poor prognosis of patients. Increased aqueous humor outflow resistance and elevated IOP caused by ECM remodeling and TM dysfunction are the most essential risk factors for glaucoma. Therefore, the genomic and RNA profiling of TM tissue-based sequencing may provide insight into understanding of glaucoma pathogenesis. We screened the overlap of miRNAs target gene and mRNA profiling of TM in POAG. GO and KEGG enrichment analyses indicated that the main signaling pathways enriched were closely associated with glaucoma advancement, which has been elucidated by previous studies. Nitric oxide biosynthetic process may contribute to regulation of ocular blood flow and AH production via cGMP pathway, based on which prostaglandin analogues are applied for glaucoma treatment [21]. Microtubule polymerization is related to ECM remodeling and TM contraction. Unfolded protein binding-mediated endoplasmic reticulum stress also plays important role in TM dysfunction and cell death of glaucoma [22]. And studies revealed that vasopressin-regulated water reabsorption can decrease IOP [23]. Then, through construction of PPI network, we screened top 30 hub target genes regulated by DEmiRNAs in TM of POAG. After verification by GSE27276 dataset, 8 genes were identified to have significant difference between controls and POAG patients, although the expression trend of some of these genes is different from our sequencing results. *HSP90AB1* was up-regulated in POAG in both GSE27276 and our dataset. *SCAMP2*, *SEMA4B* and *SLC6A4* were down-regulated in POAG in both GSE27276 and our dataset. *SLC2A3* was up-regulated in our sequencing results, but down-regulated in GSE27276. *CAPZA2*, *CREB1* and *RCOR3* were down-regulated in our sequencing results, but up-regulated in GSE27276. This may result from confounding factors such as sample source and stages of POAG. Several studies have reported the significant role of *HSP90* in glaucoma advancement [24, 25]. Recently, glaucoma was considered as a kind of autoimmune disease. And this process was correlated to heat shock protein-induced T cell response [26]. A case–control study has identified 12p13.3 copy number variation locus overlapping the genes *SLC2A14* and *SLC2A3* as a major regulator of IOP [27]. *CREB1* was also reported as a pathogenic gene involved in POAG [28]. However, the relationships between other hub genes and glaucoma are still unclear. Further studies are needed to figure out their effects on glaucoma. And 3 genes (*CREB1*, *CAPZA2*, *SLC2A3*) were obtained after diagnostic performance test. GSEA analysis indicated that these 3 genes modulated POAG through different pathways. Increasing evidences have demonstrated the relationships among *CREB1*, autophagy and POAG. Rho kinase inhibitors, ripasudil and netarsudil, have been applied as a novel therapy for glaucoma [29]. There is still little evidence showing whether *CAPZA2* and *SLC2A3* regulate advancement of POAG. While ECM remodeling and oxidative phosphorylation are classical regulatory mechanisms of POAG. A study has revealed the associations between glycosaminoglycan metabolism and POAG. They proposed that POAG might be a hyaluronic acid (a kind of glycosaminoglycan) deficiency disease [30]. In addition, alterations of the intestinal bacterial flora-induced immunological reaction are associated with POAG advancement [31]. Further studies are required to elucidate the specific regulatory mechanisms of POAG, thus providing novel insights into POAG therapy.

Clinically, there are still no definitive biomarkers for POAG. And diagnosis rests on clinical features evaluation. This leads to delayed diagnosis, especially for NTG. The stability and availability in AH, tears and plasma of miRNA make it a potential biomarker for early diagnosis of POAG. In this study, the construction of miRNA-mRNA regulatory network establishes a connection for the molecular interaction between TM and AH. Given that TM tissues are difficult to obtain for diagnosis, we further estimate the diagnostic efficacy of miRNAs in AH for POAG. 2 novel biomarkers (*miR-106b-5p*, *miR-15a-5p*) in AH exhibited a superior accuracy for diagnosis of POAG, especially combined with their target genes. Previous studies have revealed that *miR-16*, *miR-210* and *miR-637* etc. indicated a high accuracy for POAG diagnosis [32]. In our study, we didn't detect the expression of *miR-637* in both control and POAG TM samples. We have tested the accuracy of *miR-16* and *miR-210* in our dataset as shown in the following figure (Fig. S4A). And *miR-16* exhibited excellent accuracy as biomarkers of POAG. However, *miR-210* showed poor ability to distinguish POAG from non-glaucoma individuals. In early screening of POAG, patients with abnormal above indicators are recommended to conduct clinical assessment, which may help to reduce blindness caused by glaucoma. In addition, stem cell miRNA delivery therapy has been widely studied in cancer treatment research. The research and development of anti-glaucoma medications targeting miRNA are of great significance.

There are several limitations in our work. The analysis may have been limited by the small sample size. And we cannot exclude a contribution of other clinical factors to the findings, such as stages of disease and medications usage. In addition, we just verified the expression of mRNA with another dataset, but didn’t perform PCR or luciferase reporter assay. The mechanisms of how these genes regulating POAG pathogenesis are still unclear. Further studies exploring these miRNAs/mRNA axis and other possible mechanisms are needed.

## Conclusion

In brief, we integrated miRNA-mRNA regulatory network in TM and AH of POAG, and identified 2 miRNAs (*miR-106b-5p*, *miR-15a-5p*) as potential biomarkers, and 3 genes (*CREB1*, *CAPZA2*, *SLC2A3*) as driving factors for POAG. This study provides theoretical basis for pathogenesis study of glaucoma and contributes to clinical prevention, early diagnosis and individualized treatment of POAG.

### Supplementary Information

**Additional file 1:** **Figure S1.** Potential target genes of DEmiRNAs predicted by miEAA and miRNet. (A, B) Venn diagram and miRNA-target gene network indicate overlap of target genes for up-regulated DEmiRNAs predicted by miEAA and miRNet. (C, D) Venn diagram and miRNA-target gene network indicate overlap of target genes for down-regulated DEmiRNAs predicted by miEAA and miRNet. **Figure S2.** Expression levels of top 30 hub genes were indicated from the GSE27276 dataset. **Figure S3.** Identification of biomarkers for POAG in AH by ROC curves. (A~L) The potential of miRNAs in AH for identification of POAG from GSE105269 dataset. AUC at 0.5 ~ 0.7 represents low accuracy, AUC at 0.7 ~ 0.9 represents moderate accuracy, AUC above 0.9 represents high accuracy. **Figure S4.** Accuracy of *miR-16* and *miR-210* in diagnosing POAG by ROC curves.

## Data Availability

The datasets used and analyzed during the current study are available from the corresponding author on reasonable request.
