# Supplementary figures and images for: Construction of miRNA-mRNA regulatory network indicates potential biomarkers for primary open-angle glaucoma
Source: BMC Med Genomics. 2023 Nov 8;16:280. doi: 10.1186/s12920-023-01698-2 (PMC10634160; doi:10.1186/s12920-023-01698-2)

# Figure S1

A

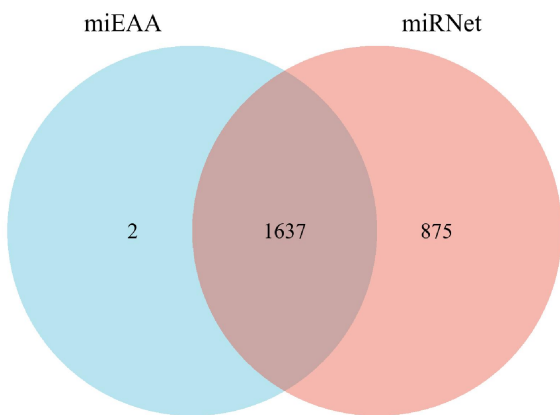

B

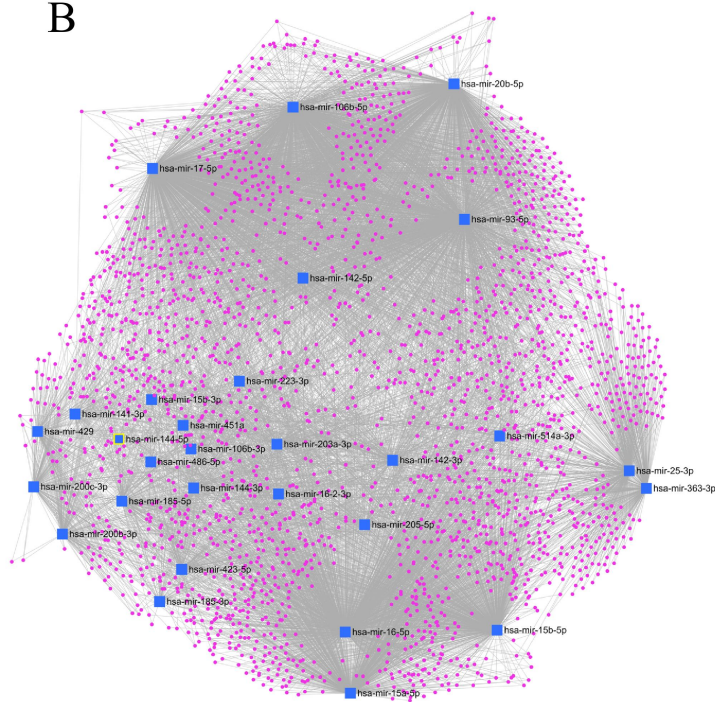

C

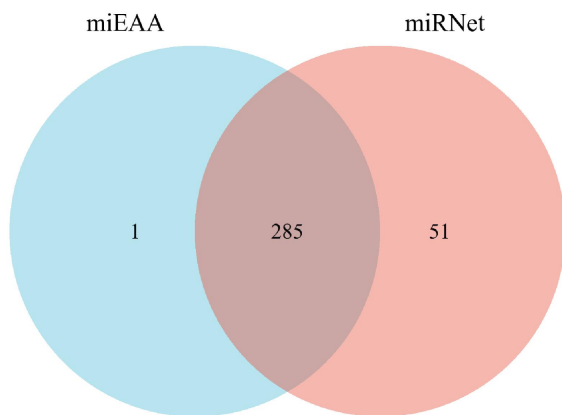

D

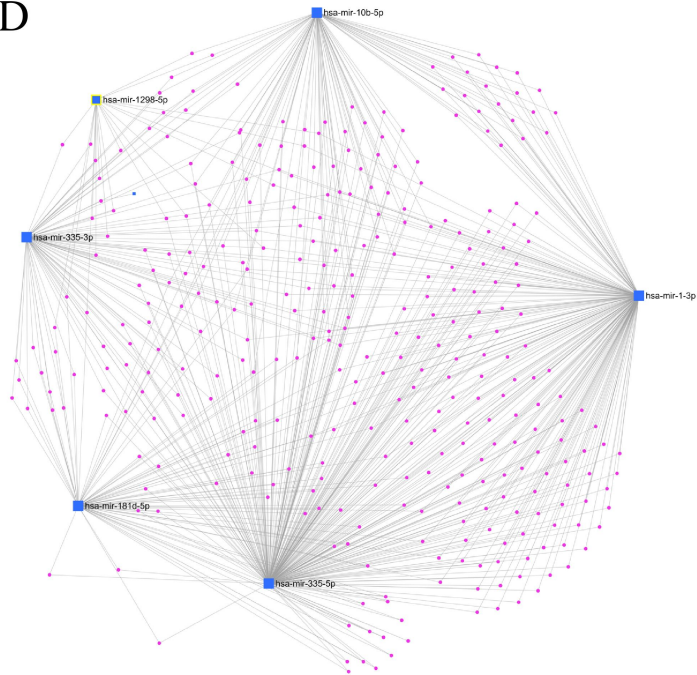

Figure S2

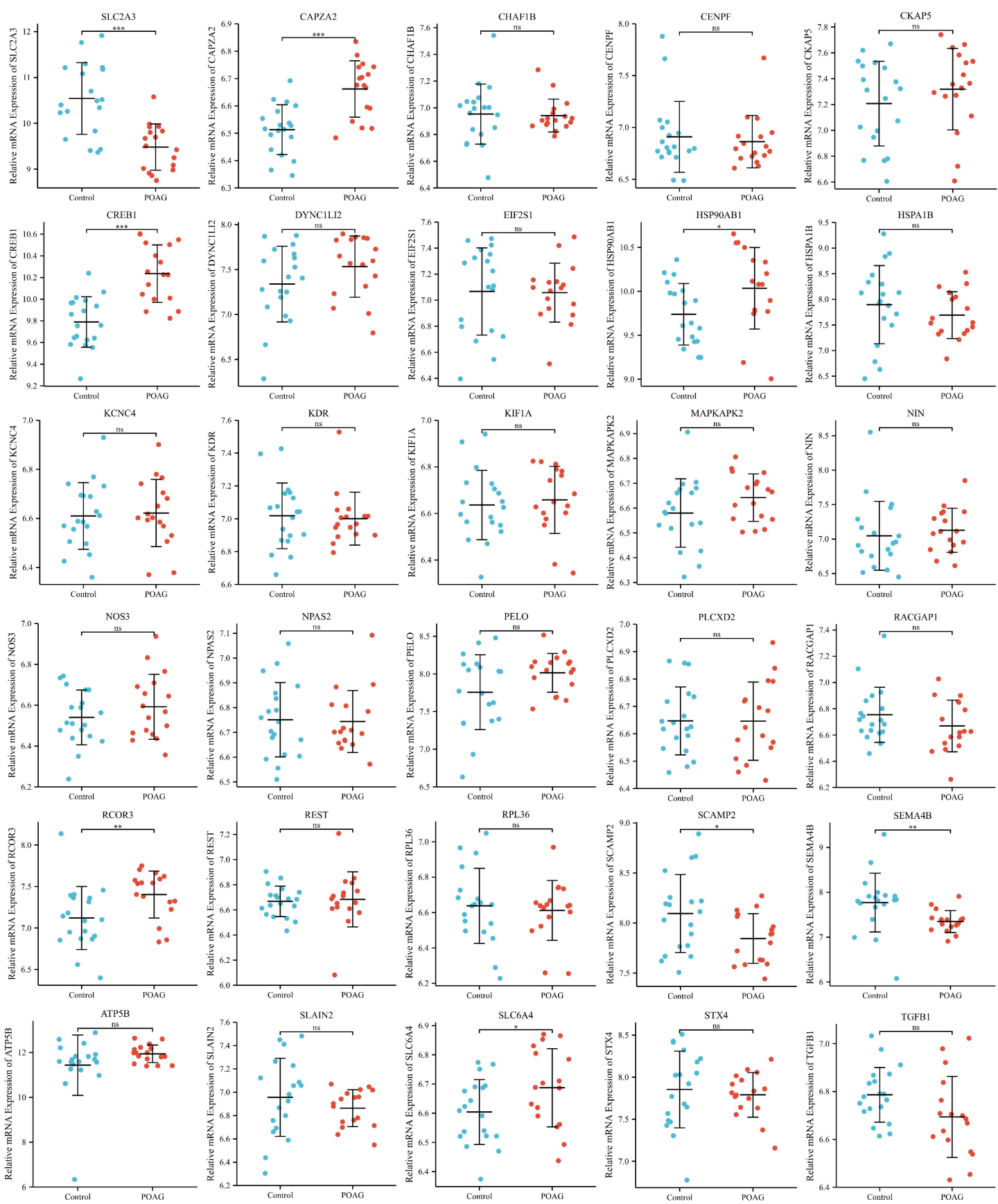

Figure S3

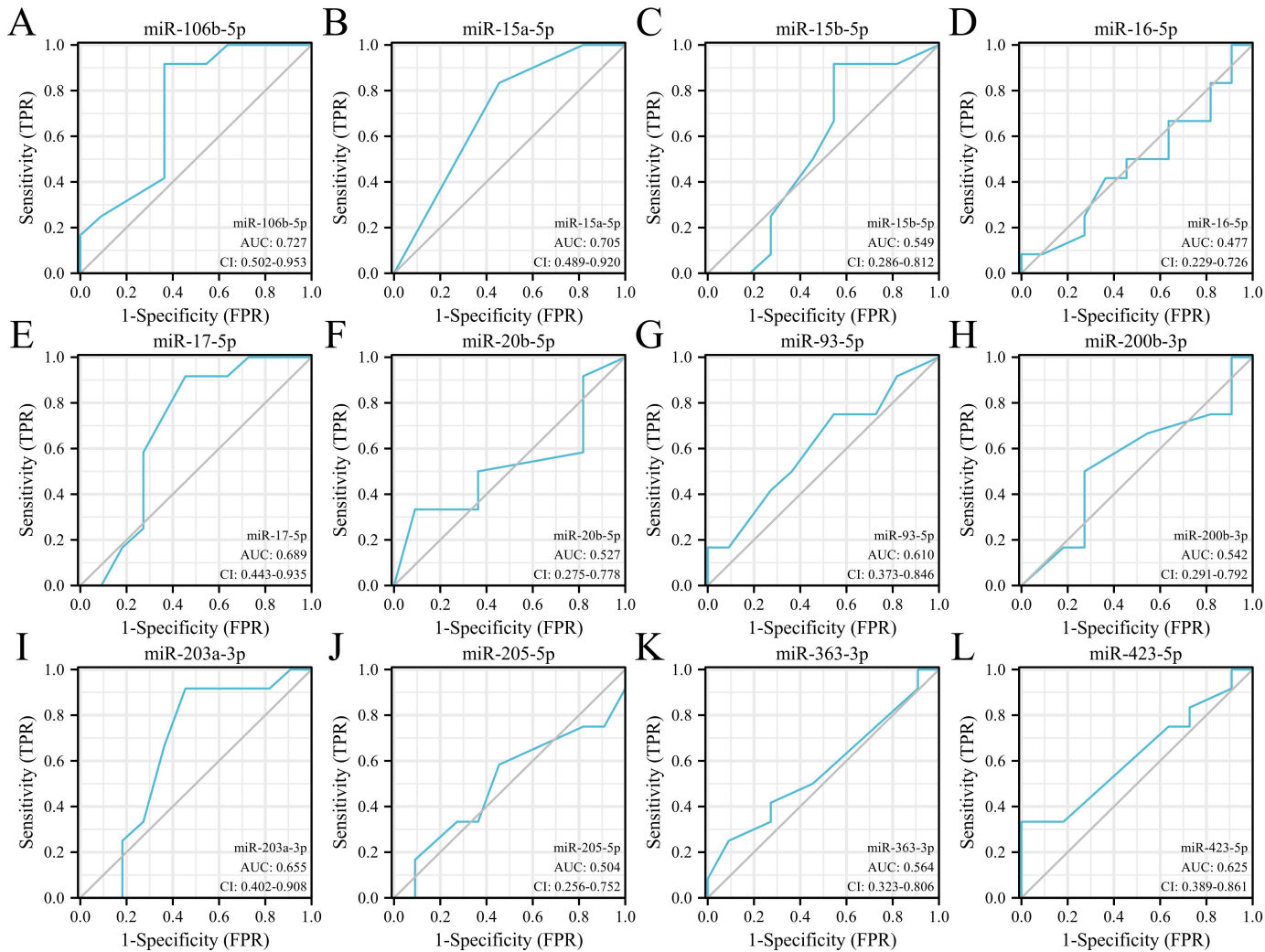

Figure S4

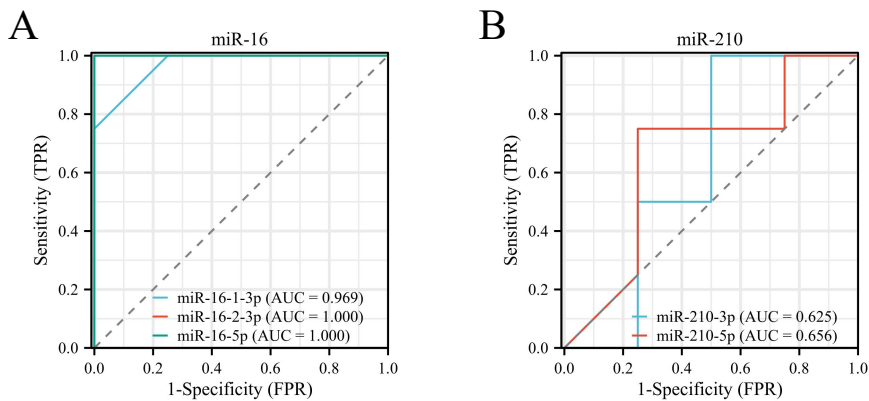

Supplement: Supplementary file 1 — Additional file 1: Figure S1. Potential target genes of DEmiRNAs predicted by miEAA and miRNet. (A, B) Venn diagram and miRNA-target gene network indicate overlap of target genes for up-regulated DEmiRNAs predicted by miEAA and miRNet. (C, D) Venn diagram and miRNA-target gene network indicate overlap of target genes for down-regulated DEmiRNAs predicted by miEAA and miRNet. Figure S2. Expression levels of top 30 hub genes were indicated from the GSE27276 dataset. Figure S3. Identification of biomarkers for POAG in AH by ROC curves. (A~L) The potential of miRNAs in AH for identification of POAG from GSE105269 dataset. AUC at 0.5 ~ 0.7 represents low accuracy, AUC at 0.7 ~ 0.9 represents moderate accuracy, AUC above 0.9 represents high accuracy. Figure S4. Accuracy of miR-16 and miR-210 in diagnosing POAG by ROC curves. [file 12920_2023_1698_MOESM1_ESM.pdf]
